# Structural and Emulsifying Properties of Citric Acid Extracted Satsuma Mandarin Peel Pectin

**DOI:** 10.3390/foods10102459

**Published:** 2021-10-15

**Authors:** Xingke Duan, Zhixuan Yang, Jinyan Yang, Fengxia Liu, Xiaoyun Xu, Siyi Pan

**Affiliations:** 1 College of Food Science and Technology, Huazhong Agricultural University, Wuhan 430070, China; Duanxk@webmail.hazu.edu.cn (X.D.); y15071358400@163.com (Z.Y.); jinyanyang@webmail.hzau.edu.cn (J.Y.); xuxiaoyun@mail.hzau.edu.cn (X.X.); pansiyi@mail.hzau.cn (S.P.); 2 Key Laboratory of Environment Correlative Dietology, Huazhong Agricultural University, Ministry of Education, Wuhan 430070, China

**Keywords:** Satsuma mandarin peel pectin, structural characterization, emulsifying

## Abstract

Satsuma mandarin peel pectin (MPP) was extracted by citric acid and its structure and emulsifying ability were evaluated. Structural characterization, including NMR, FTIR, monosaccharide compositions demonstrated that MMP showed lower DM value and higher *Mw* than commercial citrus pectin (CCP). In addition, MPP exhibited significantly better emulsification performance than CCP. When MPP concentration was increased to 1%, 1.5% (10 g/L, 15 g/L) and the pH was 3 (acidic condition), a stable emulsion containing 10% oil fraction could be obtained. The particle size of the obtained emulsion was ranging from 1.0–2.3 μm, its emulsifying activity ranged from 93–100% and emulsifying stability was 94–100%. Besides, MPP can better ensure the storage stability of higher oil ratio emulsions. The results demonstrated that the stable emulsifying properties of MPP may largely depend on the lower DM value and higher *Mw*. MPP could be used as a novel polysaccharide emulsifier, especially under acidic conditions, providing a promising alternative for natural emulsifiers that could be used in the food industry.

## 1. Introduction

Emulsifiers play a key role in the preparation of emulsions, and are important to improve the texture and taste of certain foods and beverages [1]. Certain natural polysaccharides have good emulsifying properties due to their non-polar groups or proteins attached to their hydrophilic carbohydrate chains [2]. Some polysaccharides, including certain pectins, gum arabic, and modified starches, have been shown to have expected emulsifying activity and emulsifying stability [3,4].

Pectin is a component of the plant cell wall and is a complex polysaccharide commonly found in the roots, stems, leaves, flowers, and fruits of plants [5]. It can be extracted from different sources, but commercially available pectin sources are mainly citrus peel, apple pomace, and sugar beet pulp [5,6]. The variation in pectin structure and composition results in different functionalities of which its gel-forming capacity was extensively studied in the past [7]. Recently, pectin is gaining more acceptance as an effective emulsifier in numerous food applications. In general, the emulsifying capacity of pectin is mainly attributed to the hydrophobic groups in pectin molecules such as methoxyl group, acetyl group, etc. depending on the species and chemical structure [8]. Protein is considered to play an important role in the emulsifying ability of sugar beet pectin, while methyl ester is critical for citrus pectin [9,10]. Studies of Funami [11] confirmed the key role of proteins of sugar pectin, which act as anchor for the adsorption onto the surface of oil droplets. In a study using a range of pectins from various natural sources, it was shown that small emulsion droplet sizes correlate with a comparably high protein concentration (1.1–4.7%) [9]. Once these hydrophobic groups (methoxyl group, acetyl group, protein groups, etc.) are adsorbed, pectin can promote the long-term stability of the emulsion through steric and/or electrostatic effects. The adsorbed pectin can form a thick hydration layer at the oil-water interface, causing steric hindrance and increasing the stability of the emulsion to the coalescence of droplets [12]. Studies also reported that the emulsifying ability of pectin depends on its molecular weight, RG-I ratio, and other structural characteristics. Pectins from different sources usually show very different properties due to their structural differences [11,13]. Therefore, the development of new pectin sources has obvious advantages over enzyme and chemical modification in food acceptability [14].

Citrus is currently the largest fruit crop in the world, and China has become a leading country for citrus production [15]. Mandarin (*Citrus reticulata*), the wide-skinned citrus variety, ranked the first in total citrus production in China (67%), with Satsuma mandarin (*Citrus unshiu Marc.*) being the main cultivar [16]. Except for a small amount of dried tangerine peel processing, most of citrus peel are directly discarded and buried, resulting in great waste of resources [17]. The pectin content in citrus peel is 20–30%, which is a good source of pectin [18]. Citrus pectins from different varieties showed different structural and properties. KAYA et al. [19] compared the effects of different acids on the extraction of pectin from different citrus peels. Whatever the extraction condition, grapefruit pectin samples exhibited particularly low (Ara + Gal)/Rha ratios compared with other pectins. Orange pectin samples exhibited high Rha, Gal, and Ara contents, while lemon and lime pectin samples exhibited moderate Rha, Gal, and Ara contents. Hu et al. [20] reported that the GalA content of pectin extracted from Eureka lemon, Guanxi grapefruit and grapefruit peel was significantly higher than that in navel orange peel. The number average molecular weight (Mn) of navel orange peel pectin was also the highest and showed a significant difference compared to the other cultivars. Even from the same source, different environmental conditions will affect the emulsification properties. Guo et al. [21] reported that the pomelo peel pectin emulsion showed good stability in the pH range of 3–5, and when the pH increased to 6, the emulsion stratified and could not form a stable emulsion system. Current research mainly focuses on discovering the properties of orange peel pectin, pomelo peel pectin, and lemon pectin. To the best of our knowledge, there have been very few studies concerning the structural and emulsifying properties of Satsuma mandarin peel pectin.

Thus, in this study, pectin was extracted from Satsuma mandarin peel by citric-acid extraction. The obtained pectin was then subjected to physicochemical analysis, molecular weight distribution, FTIR spectrum, and NMR spectroscopy (^1^H). Besides, to identify whether MPP could be a good emulsifier, the emulsifying properties of MPP were evaluated, commercial citrus pectin (CCP) was selected as a reference. The effects of the pectin concentration, pH and oil ratio on emulsion performance were examined. This study may provide a theoretical basis for high value utilization of satsuma mandarin peel, and facilitate the development of satsuma mandarin pectin-based emulsifier.

## 2. Materials and Methods

### 2.1. Materials

Fresh satsuma mandarin fruit was purchased from the local market (Wuhan, China). Commercial citrus pectin (CP, P9135), L-rhamnose (Rha), D-galactose (Gal), D-fructose (Fru), D-xylose (Xyl), L-arabinose (Ara), D-mannose (Man), D-galacturonic acid (GalA), D-glucuronic acid (GluA), and other standard products were purchased from Sigma-Aldrich (St. Louis, MO, USA). All chemical reagents, including ethanol, hydrochloric acid, sulphuric acid, sodium tetraborate, coomassie brilliant blue, bovine serum albumin (BSA), sodium hydroxide, trifluoraceticacid, etc., used in the experiments were analytical grade and purchased from the Sinopharm Chemical Reagent Co., Ltd. (Shanghai, China).

### 2.2. Preparation of the SAMPLE

Satsuma mandarin fruit was manually peeled and the peels were steamed at 100 °C for 5 min to destroy endogenous enzymes, and then freeze-dried with a vacuum freeze dryer. After that, the dried sample was pulverized using an electric grinder (Zhejiang Industry and Trade Co., Ltd., Zhejiang, China), and the sample was passed through a 40-mesh sieve. The Satsuma mandarin peel powder was vacuum-packed and placed in a desiccator until further use or for use.

### 2.3. Extraction of Pectin

**Figure 1:**
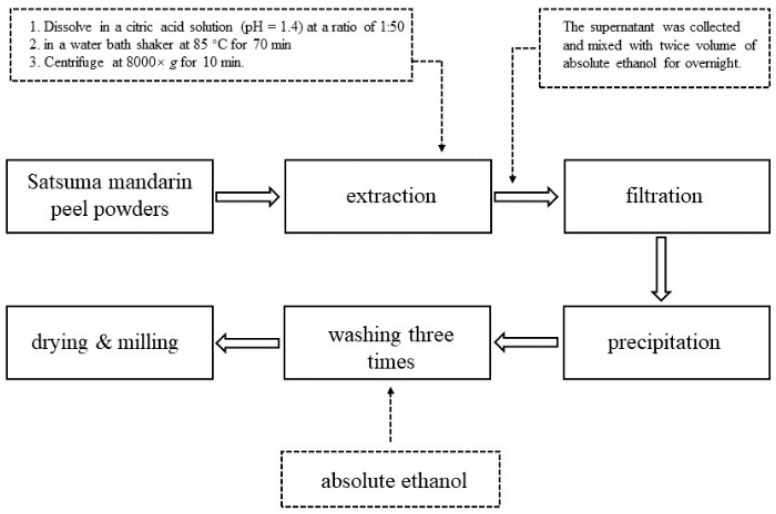
Flowchart of the pectin extraction process.

The extraction and purification of pectin was performed following the method of Guo, Zhao, Liao, Hu, Wu, and Wang [21] with some modifications. Briefly, the dry Satsuma mandarin peel powders were dissolved in deionized water at the ratio of 1:50 and then pH was adjusted to 1.4 by 1 M citric acid solution. Thereafter, the mixture was placed in a water bath shaker at 85 °C for 70 min and then cooled to room temperature. After that, the extraction solution was centrifuged at 8000× *g* for 10 min. The supernatant was collected and mixed with twice volume of absolute ethanol for overnight. The precipitated pectin was filtered through 400-mesh gauze and washed three times with 30 mL absolute ethanol, acetone, and absolute ethanol, respectively, for removing the pigment, monosaccharides, and disaccharides. Finally, the pectin was obtained by freeze-drying. A flow-diagram for the extraction procedure of the pectin is illustrated in Figure 1.

### 2.4. Structural Characteristics Determination

#### 2.4.1. Galacturonic acid (GalA) Content, Degree of Methoxylation (DM), and Monosaccharide Composition Analysis

The GalA content of pectin was measured based on the method described by Blumenkrantz and Asboe-Hansen [22]. The DM of pectin was determined based on the titrimetric method using NaOH according to the Food Chemical Codex (FCC, 2004) [23]. The monosaccharide compositions were determined based on the method of Petkowicz, et al. [24] with slight modification. MPP and CCP (5 mg) were hydrolyzed using 3 mL of 2 M trifluoroacetic acid (TFA) for 2 h at 121 °C. Then methanol was added under blowing nitrogen to remove the TFA. Before analysis, the sample after nitrogen blowing (monosaccharide after acid hydrolysis) was dissolved in ultrapure water at a ratio of 1:10, passed through a 0.22 μm ultrafiltration membrane. The HPAEC-PAD analysis was performed exactly as described in Neckebroeck, et al. [25] using a Thermo ICS5000+ system (ICS5000+, (Thermo Fisher Scientific, Waltham, MA, USA) equipped with a Dionex™ CarboPac™ PA10 (250 × 4 mm, 10 mm) and ED50 electrochemical detector (Dionex, Sunnyvale, CA, USA). The injection volume was 20 μL and the column temperature was 30 °C. The mobile phase A was H_2_O, mobile phase B was 100 mmol/L NaOH, The specific parameters of the mobile phase are shown in Table 1:

#### 2.4.2. Determination of Molecular Weight Distribution (MWD) and Average Molecular Weight (Mw)

The MWD and *Mw* values of MPP and CCP were evaluated by the gel permeation chromatography-refractive index-multiangle laser light scattering (GPC-RI-MALS) (DAWN HELEOSI, Wyatt Technology, Santa Barbara, CA, USA). The multi angle light scattering detector was DAWN HELEOS II (Wyatt technology, Santa Barbara, CA, USA), and the refractive index detector was Optilab Trex (Wyatt technology, Santa Barbara, CA, USA). The eluent was a 0.1 M NaNO_3_ containing 0.02% NaN_3_. The flow rate was maintained at 0.4 mL/min and the column temperature was set to 45 °C. The sample injection volume was 100 μL.

#### 2.4.3. Nuclear Magnetic Resonance Spectroscopy (NMR)

The ^1^H NMR spectra were obtained according to the method previously described by Xie, et al. [26]. MPP and CCP were collected in D_2_O plus 0.05% (*w*/*v*) trisodium phosphate (TSP) solvent with a concentration of 20 g/L at 298 K on a Bruker Avance III 600 MHz spectrometer (Bruker Technologies, Rheinstetten, Germany) operating at 600.12 MHz for ^1^H.

#### 2.4.4. Fourier Transforms-Infrared (FT-IR) Spectroscopy

FTIR spectroscopy measurements were determined using a Fourier Transform Infrared Spectrometer (Thermo Fisher Scientific, Madison, MA, USA), according to the method of Wan, Chen, Huang, Liu, and Pan [7]. MPP and CCP were ground into a fine powder and mixed with KBr at a ratio of 1:100. Then the mixture was placed in a dry agate mill, ground and compressed. After removal, a transparent sample sheet was obtained, and the infrared spectrum was scanned by Fourier transform infrared spectrometer with the scanning ranging from 400 to 4000 cm^−1^.

### 2.5. Emulsifying Properties

#### 2.5.1. Preparation of Emulsions

Emulsion preparation was performed based on the method of Guo, et al. [27]. After dissolving the pectin into citric acid-sodium citrate buffer solution (the pH was 3.0) to achieve a concentration gradient (0.5% (5 g/L), 1.0% (10 g/L), 1.5% (15 g/L), and 2.0% (20 g/L)), 0.05% (0.5 g/L) sodium azide was added as a preservative. Refined soybean oil (5 g Arawana, Yihai Kerry group, Shanghai, China) was mixed into the solution (15 g). Firstly, the mixture of pectin and oil was homogenized using a high-speed homogenizer (Ningbo Xinzhi Biotechnology Co., Ltd., Ningbo, China) at 10,000 rpm for 3 min to prepare coarse emulsion. Thereafter, the coarse emulsion was treated with a JY92-2D ultrasonic processor (Ningbo Sentz Biotechnology Co., Ltd., Ningbo, China) at 300 W for 10 min to obtain the final emulsion.

Besides, pectin emulsions with different pH and different oil ratios were also prepared. Pectin was dissolved in citric acid-sodium citrate buffer solutions of different pH (pH = 3, 7, 8) at a concentration of 1% (10 g/L). Then, the pectin solution (15 mL) was mixed with soybean oil (5 g) and subsequently the operation was performed as above to prepare emulsion. Similarly, emulsions (pH = 3) containing 1.0% pectin (10 g/L) and different oil ratios (10%, 25%, 50%) were prepared. Then, mixing is performed as above to prepare the emulsion. The amount of oil additions and the amount of MPP and CCP solutions to prepare the emulsion are shown in Table 2.

#### 2.5.2. Droplet Size Determination of Emulsion

Droplet size distribution was measured immediately after the emulsion preparation and after 1, 2 and 3 weeks of storage using a Malvern Mastersizer 2000 (Malvern Instruments Ltd., Worcestershire, UK) laser diffraction particle size analyzer.

#### 2.5.3. Rheological Properties of Emulsion

The apparent viscosity of pectin emulsions were obtained according to the method previously described by Yang, Nisar, Hou, Gou, Sun, and Guo [8]. The apparent viscosity was conducted using an Haake Rheostress 6000 rheometer (Thermo Scientific, New Castle, DE, USA). For entire experiment, a geometric lamina titanium alloy rotor with a cone diameter of 60 mm was used (model: C60Til, cone angle 1°, measuring distance 0.052 mm). All measurements were performed in a steady shear mode in the range 0.01–100 s^−1^ at 25 °C, and the temperature control system was Peltier system.

#### 2.5.4. Determination of Emulsion Stability

The stability of the emulsion was evaluated according to Xu, et al. [28] and Guo, Zhao, Pang, Liao, Hu, and Wu [27], and three methods were used.

A scanning light scattering instrument (Turbiscan MA 2000, Formulaction, Toulouse, France) was used for measuring the physical stability of the emulsions. 15 mL emulsion were pipetted into Turbiscan scanning tube and analyzed by a light beam emitted at a wavelength of near infrared (880 nm) that scanned the sample from bottom to top. The turbiscan scanning tube was scanned every 20 min for 10 h at 4 °C and the stability of emulsions was calculated from the changes in backscattering flux at the time of 10 h along the height of the emulsions (2–35 mm). TSI (Turbiscan index) is the evaluation index of the stability of the sample by accumulating the light intensity change value measured by the two scans at all heights of the sample. The smaller the TSI stability coefficient, the more stable the emulsion is [28].

For storage stability, the emulsion layer was measured in fresh emulsion immediately after preparation, and storage at 4 °C for 1, 2 and 3 weeks, the emulsion stability (ES) was established using the following equation ($\mathrm{ES}(\%)=\frac{\mathrm{Remained}\;\mathrm{volume}\;\mathrm{of}\;\mathrm{emulsion}\;\mathrm{layer}}{\mathrm{Initial}\;\mathrm{volume}\;\mathrm{of}\;\mathrm{emulsion}\;\mathrm{layer}}$ × 100), and then for Emulsion activity, the fresh emulsion was centrifuged for 10 min at 4000× *g* and the Emulsion activity after centrifugation (EA_10_) was established according to the equation (EA_10_ (%) = $\frac{\mathrm{volume}\;\mathrm{of}\;\mathrm{emulsion}\;\mathrm{layer}}{\mathrm{Total}\;\mathrm{volume}\;\mathrm{of}\;\mathrm{fluid}}$ × 100).

The ES_0_, ES_1_, ES_2_, and ES_3_ represent the emulsion stability for the fresh emulsion and storage at 4 °C for 1, 2, and 3 weeks, respectively.

#### 2.5.5. Morphology of Emulsion

According to the method of Bai, Huan, Li and McClements [12], a fluorescence laser microscope with a 40× objective lens (Nikon d-Eclipse C1 80i, Nikon, Melville, NY, USA) were used to examine the microstructure of the emulsion sample. The oil phase can be stained by adding 5 μL of Nile Red solution (1 mg/mL in ethanol) to 500 μL emulsion sample. After homogeneously mixing by pipette, 3–5 μL of dyed emulsion was placed on a microscope slide and covered with a glass cover slip. The cover slip was quickly fixed by nail polish to avoid evaporation. Nile Red is excited at 488 nm and emission is detected at 580 nm. All measurements are performed at 25 °C.

### 2.6. Statistical Analysis

All experiments were performed in triplicate, and results were expressed as the means ± standard deviation (SD). Analysis of variance was performed using Origin 9.0 (OriginLab Corporation, Northampton, MA, USA). The significant level was set as *p* < 0.05 throughout the study.

## 3. Results and Discussion

### 3.1. The Monosaccharide Composition, DM, and Molecular Weight of MPP and CCP

The GalA content, monosaccharide compositions, and DM of MPP and CCP are listed in Table 3. The GalA content of MPP and CCP were 72.00 ± 0.83% and 70.11 ± 0.01%, respectively. Both MPP and CCP were HMP (high methoxyl pectin), with DM > 50%. However, MPP (52.02%) showed significantly lower DM than that of CCP (67.90%), which was also lower than that of pomelo pectin (74.4%) [21] and Kara mandarin peel pectin (65.1%) [29]. DM is an interesting characteristic for emulsion stabilizing capacity of pectin, as good emulsifying properties were reported for citrus pectin with a medium DM (55%) [10]. However, Schmidt, et al. [30] stated that an increase in DM to very high value (>80%) resulted in a significant decrease of emulsion droplet sizes and improved of long-term stability. Other relevant reports stated that low DM pectin was found to reduce the interfacial tension more strongly than pectin with higher DM [31].

Mw is an indispensable factor in the study of the polysaccharide structure-function nexus [32]. The Mw of MPP was significantly higher than that of CCP, and higher than that in navel orange peel pectin (152.1 kDa) [33], grapefruit peel pectin (57.8–84.4 kDa) [34]. Previous studies suggested that short and entangled polymer chains cannot provide effective steric stability, if the Mw is too low, the adsorbed layer might be too thin to ensure sufficient stability [35,36]. In another word, higher Mw may favor emulsifying activity of pectin. The weight average molecular weight-to-number average molecular weight ratio (Mw/Mn), also known as the polydispersity index, reflects the molecular mass distribution of polysaccharides. The Mw/Mn of monodispersive polymers is 1, and higher Mw/Mn values indicate a wider molar mass distribution [37]. Compared to CPP, MPP showed a narrower molar mass distribution.

For monosaccharide content, both MPP and CCP contained an abundance of galactose (Gal), followed by the rhamnose (Rha), which was in accordance with previous results of Zhang, et al. [38]. The change of pectin main chains can be reflected by the ratio of Rha/GalA, while the change of neutral side chains of pectin are usually characterized by the ratio of (Ara + Gal)/Rha [39]. If Rha/GalA ranges from 0.05 to 1, then the main constituent of the pectin is the RG-I region [40]. In the present study, the Rha/GalA in all citrus pectins was about 0.1, which indicated high proportions of RG-I domains in MPP and CCP. Higher RG-I can also effectively promote the emulsification properties of pectin. Nakamura [41] et al. supported the positive effect of the RG-I domain on the emulsifying properties of soybean soluble polysaccharides, attributed to the ramifications (neutral sugar chains) attached to its structure, as well as the ferulic acid–arabinogalactan-protein complexes. However, no significant difference was found in the Rha/GalA ratio and (Ara + Gal)/Rha ratio of different pectins in this study.

### 3.2. Spectroscopy Analysis by NMR and FTIR

**Figure 2:**
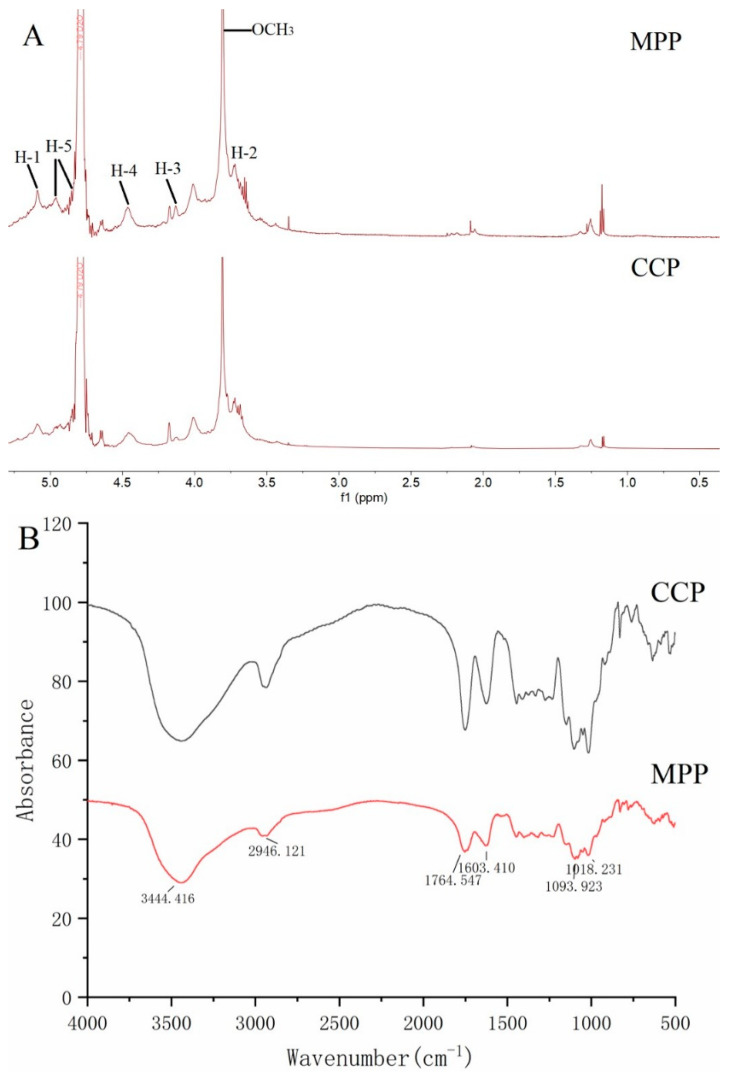
^1^H NMR spectrum (**A**) and FTIR spectrum (**B**) of MPP and CCP.

In order to analyze the structural differences of MPP and CCP, the ^1^H NMR spectrum was determined. As illustrated in Figure 2A, the most significant peak located at 4.79 ppm was attributed to the solvent signal (D_2_O). The spectrum of MPP and CCP samples contained a sharp signal at f1 = 3.81 ppm, which corresponded to the proton in the methoxyl group of the esterified pectin [42]. The signals of 4.9 (H-5) and 5.0 (H-1) ppm belong to non-esterified galacturonic acid units. The H-1, H-2, H-3, and H-4 proton signals of galacturonic acid and methyl galacturonic acid residues were observed at 5.1, 3.7, 4.1, and 4.5 ppm, respectively [43]. As compared to CCP, two relatively weak signals at 2.07 and 2.18 ppm in MPP may be due to the acetyl esterified carboxyl groups of GalA units [44]. According to the study of Leroux, Langendorff, Schick, Vaishnav, and Mazoyer [36], the higher the degree of acetylation, the better the emulsification of citrus pectin.

The FT-IR spectra of pectin are shown in Figure 2B. The broad peaks appearing between 3600 and 3200 cm^−1^ were the result of the O-H stretching vibration, indicating the presence of intra-molecular and inter-molecular hydrogen bonds. The medium absorption peaks approximately 2946 cm^−1^ were attributed to the C-H stretching of CH_2_ groups [26]. Both pectin samples had two important bands at 1756 cm^−1^ and 1603 cm^−1^ assigned to the methylesterified carbonyl groups (C=O) and the ionic carboxyl groups (COO^−^), respectively. The DM of pectins can be determined based on the ratio between the area of the 1756 cm^−1^ to the total area of the 1756 and 1603 cm^−1^ region. Both MPP and CCP were HMP, which were in agreement with the values obtained by titration. The signals in range 950–1250 cm^−1^ were possibly related to C-O-C glycoside bonds presented in the pyranose ring [45]. Similar bands were observed in pectin extracted from lime peel [46], pomelo peel [21] and Kara mandarin peel [29]. By comparing the changes of MPP and CCP, both pectins showed a similar transmission mode and the difference lied in the strength of each absorption peak.

### 3.3. Emulsifying Properties

#### 3.3.1. The Particle Size

The average particle size (D_4,3_) of freshly prepared emulsions and emulsions during storage are exhibited in Table 4, Table 5 and Table 6. When pectin concentration was 0.5%, the D_4,3_ of MPP and CCP emulsions were the largest, respectively 4.2 ± 0.3 μm and 19.7 ± 3.3 μm. As the concentration of pectin increased, the D_4,3_ of the MPP emulsion gradually decreased, but there was no significant difference between 1.5% and 2%. Similar results was reported by Liu, Pi, Guo, Guo, and Yu [14], the D_4,3_ reached its minimum when the concentration of beet pectin was 1.5%. These results could be because when pectin adsorption concentration increased to a certain value, the surface of the emulsion oil droplets was completely covered by the emulsifier, and the emulsion particle size was also reduced to a relatively stable value [1]. After being placed at 4 °C for 3 weeks, the D_4,3_ of each emulsion increased, especially for 0.5% CCP emulsion, which increased from 19.7 ± 3.3 μm to 88.3 ± 0.8 μm. In a word, whether it was freshly prepared or during storage, the D_4,3_ of MPP emulsion was significantly smaller than that of CPP, regardless of pectin concentration. It can be considered that the emulsification performance of MPP in studied concentrations were significantly better than that of CCP.

For fresh emulsion, the D_4,3_ of MPP and CCP emulsions were the smallest at pH = 3 (acidic condition), which were 2.1 ± 0.0 μm (MPP) and 13.9 ± 1.3 μm (CCP), respectively. When pH rose from 3 to 7 (neutral condition), the D_4,3_ of the two emulsions increased, which implied a better emulsifying capacity of all studied pectin at acidic pH. These results may be due to the better adsorption of pectin at the interface at acidic pH, resulting in a denser structure, which better stabilizes the newly formed interface during the emulsification process [47]. At higher pH (7, 8), the pectin conformation is more extended as almost all carboxyl groups are ionized. Consequently, more inter- and intramolecular repulsion occurs, which can lead to fewer groups adsorbing at the oil-water interface [13]. After being placed at 4 °C for 3 weeks, the D_4,3_ of all emulsions increased. In particular, the D_4,3_ of CCP emulsion with pH = 3 increased significantly during the first week. However, there was no significant change in the D_4,3_ of MPP emulsion (pH = 3) throughout the storage period, and the D_4,3_ of MPP emulsion was always smaller than that of CCP emulsion at studied pH. Therefore, it can be considered that the MPP emulsion in acid pH (pH = 3) could be potentially used as an emulsifier exhibited better storage stability.

The D_4,3_ of all emulsions increased as oil ratio increased. When oil ratio was 10%, both of them reached the smallest. This was because when the volume fraction of oil phase increased, there were fewer emulsifier molecules adsorbed on the interface of the oil droplets, which were insufficient to form stable emulsion particles, and cannot effectively prevent the mutual aggregation of oil droplets [48,49]. Similarly, an increase in rice bran oil concentration led to an increase in the size of emulsion droplets, which was due to the increase in the number of internal phases of the emulsion droplets [48]. After being placed at 4 °C for 3 weeks, the D_4,3_ of all emulsions increased. When oil ratio was 10%, the D_4,3_ of two emulsions was the smallest and there were no significant changes during 3-week storage, which indicated that MPP and CCP emulsions can maintain good storage stability in lower oil phase. Interestingly, when oil ratio increased (25%, 50%), D_4,3_ of MPP emulsion did not change significantly with the extension of storage time, while that of CCP emulsion increased, which was significant in the first week. It can be concluded that MPP can better ensure the storage stability of higher oil ratio emulsions.

#### 3.3.2. The Stability of Emulsion

**Figure 3:**
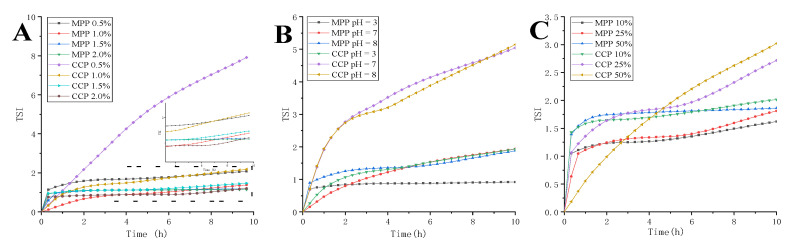
The effect of different pectin concentration (**A**), pH (**B**) and oil ratio (**C**) on the TSI (Turbiscan index) of the emulsions.

Multiple light scattering technology can promote fast and effective evaluation of fluid stability [50]. As shown in Figure 3A, the TSI of different emulsion decreased as pectin concentration increased from 0.5% to 2%, which indicated improved stability. The increase of pectin concentration produced a lower TSI, which was attributed to small size of particles that were associated to more stable emulsions. When MPP and CCP concentration increased to 2%, the TSI reached the lowest value. The TSI of MPP was always lower than that of CCP, when the pectin concentration was 0.5–1.5%, which indicated that MPP could prepare a more stable and uniform emulsion. As pH increased, the TSI of emulsion increased significantly, indicating that the stability of emulsion decreased with the increase of pH. The TSI of MPP emulsion at different pH was significantly lower than that of CCP emulsion. According to the trend of slope change, the TSI of MPP emulsion tended to be stable, meaning that the emulsion gradually stabilized. The TSI of two pectin emulsions was the smallest when pH = 3 (acidic condition), indicating the stability of the emulsion was the best at studied acidic condition. Moreover, as oil ratio increased, the TSI of emulsion increased significantly, indicating that the stability of emulsion decreased with the increase of oil ratio. It can be observed that the TSI of MPP under different oil ratio was lower than that of CCP, even if the MPP emulsion contained 50% oil phase, the TSI was still significantly lower than that of CCP emulsion with 10% oil phase. These results indicated that the MPP emulsion exhibited more stable physical properties.

When subjected to large centrifugal force, the slow increase of the instability index indicated the good stability of emulsion against creaming [25]. After centrifugation under 4000× *g* for 10 min, no delamination occurred in the emulsions with concentration of 1.5% and 2.0% of MPP, but different proportions of delamination occurred in the emulsions with concentration of 0.5% and 1.0%. However, CCP emulsions with different pectin concentrations (0.5–2.0%) exhibited different degrees of stratification after centrifugation. Compared with pH = 7 (neutral conditions) or 8 (alkaline conditions), MPP emulsion was more stable under pH = 3 (acidic condition), with EA_10_ = 93.21%, and similar results were observed in CCP emulsion. In addition, the EA_10_ decreased as oil ratio increased, which indicated that the increase in oil ratio reduced the stability of the pectin emulsion. The EA_10_ of MPP emulsion was higher than that of CCP. These results fully proved that MPP showed better centrifugal emulsification stability.

**Figure 4:**
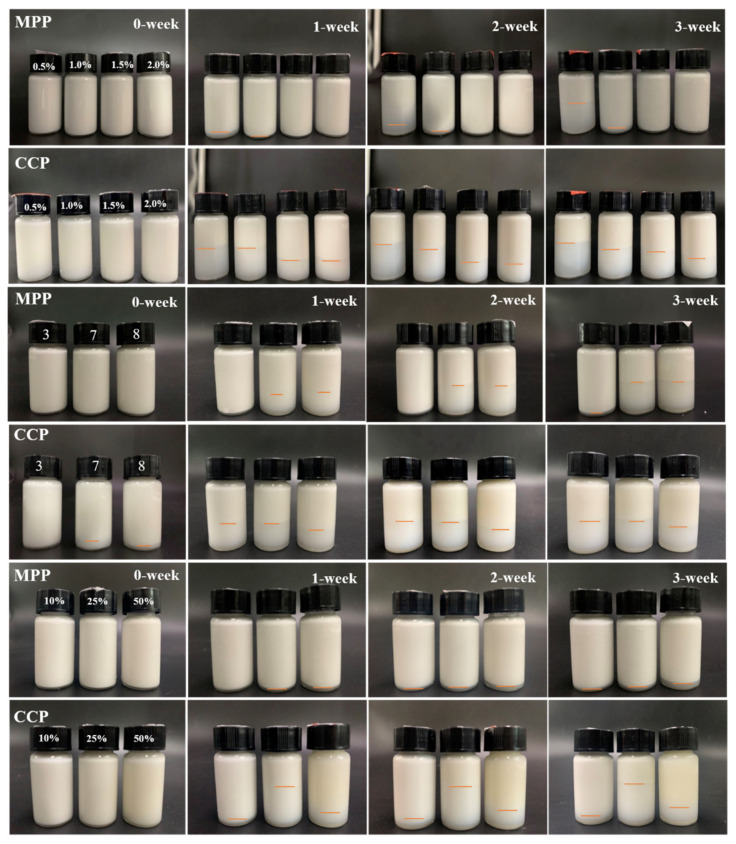
Macroscopic pictures of emulsions prepared by MPP and CCP over 3-week storage. A scanning light-scattering instrument was used to test the physical stability of the emulsion. Moreover, centrifugal determination and storage evaluation, assisted by the macro photos during the storage process, comprehensively evaluated the emulsion stability.

The storage stability of emulsions can be evaluated through the results exhibited in Table 7 and Figure 4. MPP emulsion did not show demulsification or phase separation within 24 h. After 1-week storage, MPP emulsions with pectin concentration of 0.5% and 1.0% began to delaminate, while those of 1.5% and 2% showed no obvious phase separation through 3-week storage, and the ES_3_ was 100%. It basically conformed to the law that the higher the concentration of emulsifier was, the higher ES it got. Such conclusions were in agreement with the observations of Jamsazzadeh Kermani, et al. [51] for mango pectin. As a comparison, emulsion containing 0.5% and 1% CCP exhibited rapid phase separation within 24 h, and all emulsions showed different degrees of phase separation during 3-week storage. These results intuitively showed that the storage ability of MPP was significantly better than that of CCP.

For fresh MPP emulsion, a uniform milky white color was obtained at different pH and no delamination occurred. The emulsions with pH = 7 and 8 began to separate after 1-week storage. Only a small amount of phase precipitation occurred in the emulsion with pH = 3 after 3 weeks, with ES_3_ being 96.58%. For CCP emulsions, the emulsions with pH = 7 and 8 had already exhibited obvious phase separation within 24 h, and CCP emulsions with different pH had a cream layer within 3 weeks of storage. The ES_3_ (96.58%) of MPP with pH = 3 was the highest, and significantly higher than that in CPP emulsion at any pH (pH = 3, 55.65%; pH = 7, 58.52; pH = 8, 64.38%). These phenomena indicated that the storage stability of MPP emulsion with pH = 3.0 was the highest. As exhibited in Figure 4, only a small amount of phase precipitation occurred in all MPP emulsions after 3 weeks, and ES_3_ was between 92% and 100%, which indicated that MPP emulsions can maintain uniformity and stability under different oil ratios. As a comparison, there was no significant difference in the ES_3_ of the CPP emulsion when oil ratio was 10% and 50%, and a small amount of stratification occurred during 3-week storage. However, significant stratification occurred in CPP emulsion with oil ratio of 25% during storage. Based on the above results in TSI value, the TSI of CCP emulsion with 10% oil ratio was significantly lower than that in 50% emulsion, indicating that emulsion with 10% CCP showed better emulsification stability. Therefore, these phenomena indicated that the storage stability of MPP and CCP emulsion with 10% oil ratio was the best, and the storage stability of the MPP emulsion was better than that of the CCP.

#### 3.3.3. Viscosity of Emulsion

In all cases, a non-Newtonian shear thinning behavior was found in this study, which was in agreement with the behavior reported for pectin emulsion from different botanical origins [52].

**Figure 5:**
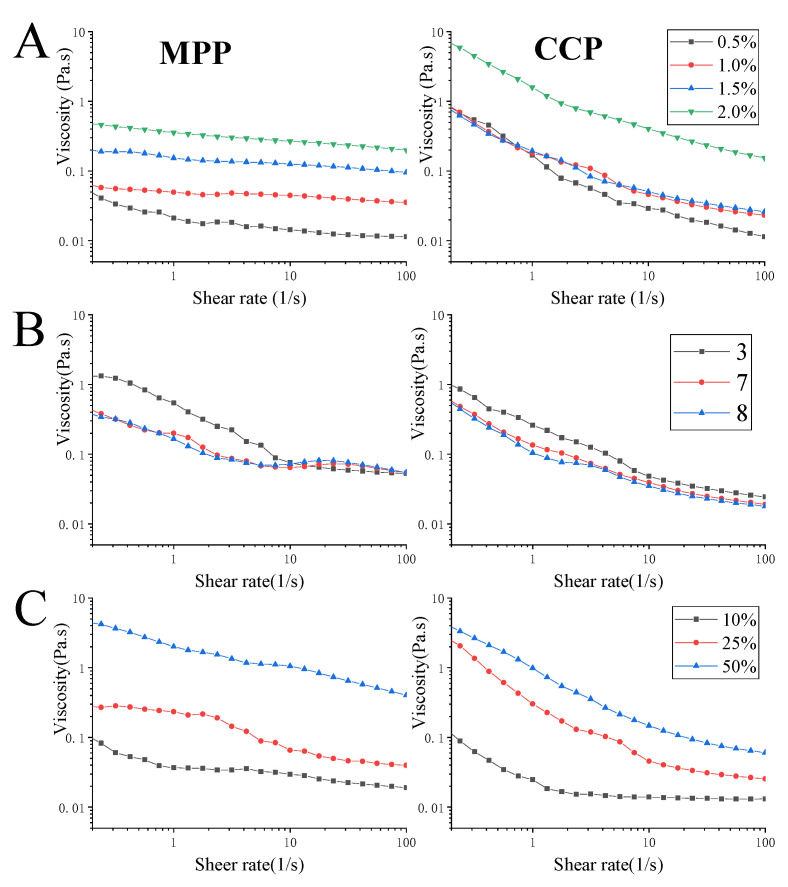
The effect of different pectin concentration (**A**), pH (**B**) and oil ratio (**C**) on the apparent viscosity of emulsion.

In a certain concentration range (0.5–2.0%), the viscosity of the emulsion increased with the increase of different pectin concentration (Figure 5A). This finding was consistent with the polysaccharide emulsion results reported by Zhao, et al. [53]. This was because as pectin concentration increased, the aggregation effect of pectin molecules was enhanced, and the strong electrostatic repulsion effect caused the pectin molecules to extend in the chain direction, which effectively promoted the cross-linking between pectin molecules [52,54]. Generally, higher emulsion viscosity may result in higher emulsion stability. However, the stability of emulsion was also affected by the particle size of the emulsion, the creaming rate was proportional to the square of the droplet radius [14]. There was no significant difference in D_4,3_ of the emulsion, when concentration was 1.5–2.0%, indicating that the emulsification rate of the pectin emulsion was not significantly changed. Besides, all emulsion was prepared by ultrasonic emulsification. The principle of ultrasonic emulsification was to divide large droplets into small droplets through cavitation, and the bubbles induced by the cavitation droplets flowed in the emulsion to make the emulsion uniform. Too high solution viscosity may limit the mixing efficiency and make the input energy transmission more uneven [49,55]. Therefore, it is speculated that the pectin concentration of 0.5% and 2% may not be suitable for emulsion preparation and 1% and 1.5% concentration could prepare emulsions with better emulsifying activity and stability.

With the increase of pH (3, 7, 8), the apparent viscosity of MPP and CCP emulsions decreased significantly (Figure 5B). MPP and CCP emulsions exhibited higher viscosity under acidic pH (pH = 3), and the viscosity curves almost overlap at pH 7 and 8. The association state of hydrogen bonds in pectin solution changed with the change of pH, which affected the apparent viscosity of pectin. When pH was higher than 3.5, the carboxyl groups of pectin were ionized. Due to the electrostatic repulsion between the carboxylate anions, the biopolymer chain can be extended [13]. It has been shown that okra and sugar beet pectin stabilize o/w interfaces at low pH values, where biopolymers adopt highly compact conformations resulting in the formation of thick interfacial layers and higher viscosity, thus providing effective steric stabilization [56,57]. In addition, at lower pH (pH = 3), the strong electrostatic repulsion between pectin molecules caused the viscosity of the continuous phase to increase, thereby reducing the possibility of droplet collisions. Therefore, the particle size of the emulsion droplets was small and not easy to aggregate, promoting better emulsification activity and stability of the pectin emulsion [3].

At the same pectin concentration (1%, 10 g/L), the increase in oil phase ratio significantly increases the apparent viscosity of different pectin emulsions, which should be expected because the increase in the content of the dispersed phase will lead to an increase in the viscosity of the emulsion. When oil ratio was high, the strong shear stress may cause the rearrangement of the emulsion droplets, which in turn enlarged the steric hindrance effect and increased the intermolecular friction of the pectin, and ultimately led to an increase in the apparent viscosity [8]. This result was consistent with studies on persimmon peel pectin [52] and pomegranate peel pectin [8]. Higher emulsion viscosity may result in higher emulsion stability. However, the influence of oil content on emulsion stability needed to be viewed dialectically. As mentioned above, the D_4,3_ of MPP and CCP emulsion increased with the increase of oil ratio, which may promote the acceleration of emulsification and reduce the stability of the emulsion. Therefore, it is necessary to combine the analysis of Section 3.3.2 to comprehensively study the stability of the emulsion.

More methods are needed to comprehensively study the stability of emulsions.

#### 3.3.4. Microstructural Observations

**Figure 6:**
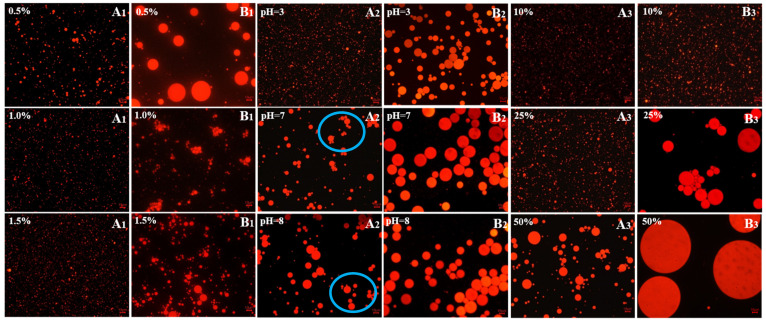
Digital photos of emulsions stabilized by MPP (**A_1_**,**A_2_**,**A_3_**) and CCP (**B_1_**,**B_2_**,**B_3_**). (**A_1_**,**B_1_**): different pectin concentration; (**A_2_**,**B_2_**): different pH; (**A_3_**,**B_3_**): different oil ratio.

**Figure 7:**
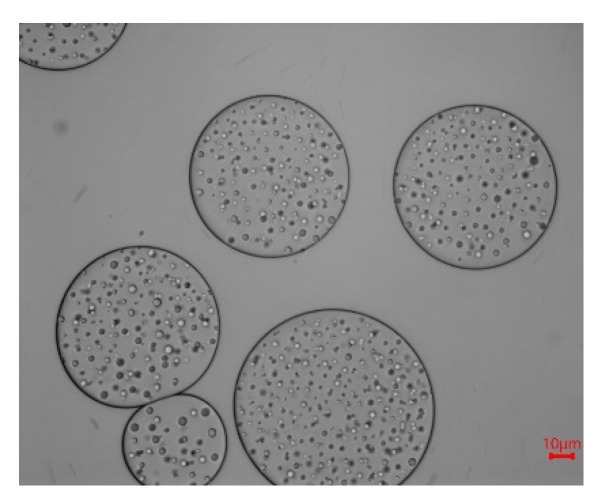
Digital photos of emulsions stabilized by CCP (oil ratio was 50%).

The typical micrographs for emulsions prepared by MPP and CCP at different concentration, pH, and oil ratio are presented in Figure 6. The droplet size decreased with increase of MPP and CPP pectin concentration. Compared with CPP, the MPP emulsion showed relatively small particle size. When pH was 3, MPP emulsion showed a uniform and relatively small droplet size. As pH value increased, the droplets flocculated and coalesced. Both for MPP and CCP, there was no significant difference in the particle size of the emulsion droplets at pH 7 and 8, which confirmed the results of the previous particle size and stability measurement. In addition, when increasing the oil ratio, the particle size of the emulsion prepared by MPP and CCP increased. What’s interesting is that when the oil ratio was 50%, the droplet size increased significantly (71.8 ± 6.9 μm), an adsorption film was formed around the droplet, and it could be seen that the surface of the droplet is not completely wrapped. Some literature refers to this similar structure as the “gingerbread” structure [10]. Figure 7 showed unstained pictures of the same sample at the same multiple, to make it easier to see. Previous studies reported that in the presence of monovalent and divalent cations, pectin with low DM may form a firm gel and a similar “gingerbread” structure could appear [10]. However, in this study, there were no cations in emulsion system and the volume of hydrocolloid emulsifier might have been too low. But if the oil content was too high, the constant pectin content is not enough to cover the oil droplets [58]. As a result, there may be exposed patches of oil droplets, which greatly increased the likelihood of coalescence [59].

### 3.4. Relation between Emulsion Properties and Pectin Structure

The emulsifying capacity of pectin is typically associated with the chemical structure of biopolymer backbone such as the DM and degree of acetylation, and the macromolecular characteristics of pectin chains (Mw, RG-I, hydrodynamic volume) [8,35,36]. In this study, MPP showed significantly lower DM (52.02%) than that of CCP (67.90%), and MPP showed significantly higher Mw (294.25 kDa) than CCP (255.95 kDa). The impact of Mw and DM on emulsifying properties of pectin has been widely reviewed in the past. DM is an interesting characteristic for the emulsion stabilizing capacity of pectin polymers. Yapo et al. [60] stated that low DM pectin was found to reduce the interfacial tension more strongly than higher DM pectin. However, these results contradicted the findings of Schmidt, Koch, Rentschler, Kurz, Endreß, and Schuchmann, [30] who stated that increasing DM from ~70% to ~80% improved the emulsification ability of citrus pectin. Interestingly, it has been also shown that increase of DM beyond 80% did not result in further reduction of droplet size something that has been attributed to the self-association of citrus pectin and, therefore, decrease in the accessibility of hydrophobic groups to the oil-water interface. Verkempinck et al. [47] reported better emulsifying potential for high DM citrus pectin (DM = 84) in comparison to medium and low DM citrus pectin (DM = 55, 70). Other authors investigated citrus pectin with DM ranging from 22 to 73% and concluded that the content of methyl esters is of minor importance for the emulsifying properties pectin [35]. The effect of pectin DM on emulsification characteristics needs further study.

In addition, it has been reported that higher Mw pectin exhibited better emulsion stability. The cross-linking of ferulic acid groups increased the Mw of sugar beet pectin, the emulsion prepared with cross-linked biopolymer (Mw~1860 kDa) had a smaller D_4,3_ and improved long-term stability [61]. Besides, pectin fractions of very low Mw result in lower interfacial activity and coarser emulsions due to the inability of short, disentangled polymer chains to provide efficient steric stabilization [31,35]. However, some articles also reported inconsistent results. Compared with higher Mw (562, 470, 282 kDa), beet pectin with lower Mw (153, 155, 306 kDa) result in formation emulsion with a larger D_4,3_, and a better storage stability [62]. It may be because the protein and/or ferulic acid of pectin are integrated with Mw to affect the emulsification properties [63]. The Mw of potato pectin decreased due to high pressure treatment, and the pectin emulsion exhibited increased viscosity and improved emulsifying properties [26]. Other studies showed that pectin with reduced Mw neither significantly reduce droplet size nor improve emulsion stability [30].

As discussed above, MPP and CCP showed similar monosaccharide composition, FTIR and ^1^H NMR spectroscopy, and all pectins belong to HMP. It can be inferred that the emulsification performances of MPP are better than CCP under different conditions because of the lower DM and higher Mw.

## 4. Conclusions

In the present study, pectin was extracted from the Satsuma mandarin peel by citric acid and its structural and emulsifying properties was evaluated. The obtained MPP was rich in galacturonic acid (72.00%) and showed a DM of 52.02%, which was significantly lower than DM of other citrus pectin, including CCP. Compared with CCP, MPP showed a higher Mw (294.25 kDa). By assessing the emulsifying properties, MPP emulsion exhibited more physical, centrifugal and storage stability, and both emulsifying activity and emulsifying stability of MPP were affected by pectin concentration, pH value, and oil ratio. When the MPP concentration in the emulsion was 1%, 1.5% (10 g/L, 15 g/L), acidic condition (pH 3), and 10% oil ratio, the emulsion exhibited the best emulsification performance. These results demonstrated that Satsuma mandarin peel could be a promising source of pectin and the excellent emulsifying properties of MPP may largely depend on the lower DM and the higher *Mw*. MPP can be used as a new polysaccharide emulsifier in the food industry, especially under acidic conditions. The study may provide some reference significance for some applications of emulsion. However, the real system is more complicated, and the application of pectin emulsifier in the real system needs further research.

## Figures and Tables

**Table 1:** Mobile phase gradient.

	Time (min)	Flow Rate (min)	A Phase (%)	B Phase (%)
Mobile phase gradient	0.0	0.5	97.5	2.5
	30.0	0.5	80.0	20.0
	30.1	0.5	60.0	40.0
	45.0	0.5	60.0	40.0
	45.1	0.5	97.5	2.5
	60.0	0.5	97.5	2.5

**Table 2:** The amount of oil additions and the amount of MPP and CCP solutions.

Oil Ratios	The Amount of Oil Added (g)	CCP/MPP Solution (g)
10%	2	18
25%	5	15
50%	10	10

**Table 3:** The monosaccharide composition, DM, and molecular weight of MPP and CCP.

		MPP	CCP
	GalA (%)	72.0 ± 0.8 ^a^	70.1 ± 0.0 ^a^
	DM (%)	52.0 ± 0.8 ^b^	67.9 ± 1.6 ^a^
Relative monosaccharide content (%, *w*/*w*)	Fuc	0.4 ± 0.0 ^a^	0.2 ± 0.0 ^b^
	Rha	6.3 ± 0.2 ^a^	6.5 ± 0.0 ^a^
	Ara	7.5 ± 0.1 ^a^	3.2 ± 0.0 ^b^
	Gal	9.0 ± 0.3 ^b^	14.0 ± 0.1 ^a^
	Glc	1.6 ± 0.0 ^b^	4.3 ± 0.1 ^a^
	Xyl	1.3 ± 0.0 ^a^	0.6 ± 0.0 ^b^
	Man	0.7 ± 0.1 ^a^	0.2 ± 0.0 ^b^
	Fru	0.6 ± 0.1 ^a^	0.5 ± 0.1 ^a^
Monosaccharide ratio	Rha/GalA	0.1 ± 0.0 ^a^	0.1 ± 0.0 ^a^
	(Gal + Ara)/Rha	2.6 ± 0.0 ^a^	2.6 ± 0.0 ^a^
Molecular weight (kDa)	Mw (kDa)	294.3 ± 2.5 ^a^	256.0 ± 4.7 ^b^
	Mn(kDa)	125.1 ± 7.1 ^a^	97.6 ± 2.3 ^b^
	Mw/Mn	2.4 ± 0.1 ^a^	2.6 ± 0.0 ^b^

GalA, galacturonic acid; DM, degree of methoxylation; Fuc, fucose; Rha, rhamnose; Ara, arabinose; Gal, galactose; Glc, glucose; Xyl, xylose; Man, mannose; Fru, fructose; Mw, average molecular weight; Mn, number average molecular weight. Note: different letters denote significantly different in the row (*p* < 0.05).

**Table 4:** The effect of different pectin concentration on the average particle size (D_4,3_, μm) of pectin emulsion.

		0		1-Week		2-Week		3-Week	
		MPP	CCP	MPP	CCP	MPP	CCP	MPP	CCP
Pectin concentration	0.50%	4.2 ± 0.3 Ae	19.7 ± 3.3 Ad	6.4 ± 0.3 Ae	50.2 ± 1.5 Ac	6.3 ± 0.6 Ae	68.1 ± 2.6 Ab	8.3 ± 2.4 Ae	88.3 ± 0.8 Aa
	1.0%	2.3 ± 0.1 Be	14.7 ± 0.4 >Bd	2.8 ± 0.2 Be	21.9 ± 1.3 Bc	2.8 ± 0.4 Be	23.0 ± 0.6 Bb	2.8 ± 0.2 Be	24.2 ± 0.3 Ba
	1.50%	1.7 ± 0.1 Cd	12.3 ± 2.3 Bc	1.8 ± 0.2 Cd	14.6 ± 1.5 Ca	1.8 ± 0.1 Cd	17.5 ± 1.2 Cbc	1.9 ± 0.1 Cd	15.8 ± 3.3 Cab
	2.00%	1.5 ± 0.2 Cd	12.9 ± 1.1 Ba	1.5 ± 0.1 Cd	12.6 ± 0.5 Da	1.5 ± 0.1 Cd	13.0 ± 1.0 Db	1.5 ± 0.1 Cd	10.3 ± 0.4 Dc

Note: different uppercase letters denote significant difference in the column and different lowercase letters denote significant difference in the row (*p* < 0.05).

**Table 5:** The effect of different pH on the average particle size (D_4,3_, μm) of pectin emulsion.

		0		1-Week		2-Week		3-Week	
		MPP	CCP	MPP	CCP	MPP	CCP	MPP	CCP
pH	3	2.1 ± 0.0 Ad	13.9 ± 1.3 Ac	2.3 ± 0.0 Ad	23.8 ± 0.3 Aab	2.8 ± 0.7 Ad	23.3 ± 0.9 Ab	2.2 ± 0.1 Ad	24.3 ± 0.4 Aa
	7	8.61 ± 1.3 Be	18.8 ± 0.9 Bc	9.7 ± 0.4 Bed	23.8 ± 0.2 Ab	9.6 ± 0.4 Bed	23.6 ± 0.0 Ab	10.9 ± 1.2 Bd	25.7 ± 0.9 Aa
	8	8.4 ± 0.3 Bf	19.4 ± 0.4 Bc	10.1 ± 0.3 Bd	23.9 ± 0.4 Ab	9.4 ± 0.0 Bd	24.2 ± 0.5 Ab	10.3 ± 0.1 Bd	26.3 ± 0.2 Aa

Note: different uppercase letters denote significant difference in the column and different lowercase letters denote significant difference in the row (*p* < 0.05).

**Table 6:** The effect of different oil ratio on the average particle size (D_4,3_, μm) of pectin emulsion.

		0		1-Week		2-Week		3-Week	
		MPP	CCP	MPP	CCP	MPP	CCP	MPP	CCP
Oil ratio	10%	1.0 ± 0.0 Ad	2.0 ± 0.1 Ad	1.4 ± 0.4 Ad	3.2 ± 0.2 Aa	1.5 ± 0.4 Ad	4.2 ± 1.2 Ac	1.3 ± 0.4 Ad	5.5 ± 0.5 Ab
	25%	2.2 ± 0.1 Bd	14.8 ± 2.1 Bc	2.6 ± 0.1 Bd	23.4 ± 1.1 Bb	2.7 ± 0.4 Bd	27.4 ± 0.9 Ba	2.4 ± 0.1 Bd	26.1 ± 0.2 Ba
	50%	7.7 ± 0.3 Ce	41.6 ± 0.9 Cc	8.9 ± 0.1 Cde	65.9 ± 2.3 Cb	11.1 ± 0.2 Cde	67.7 ± 2.8 Cab	12.8 ± 0.1 Cd	71.8 ± 6.9 Ca

Note: different uppercase letters denote significant difference in the column and different lowercase letters denote significant difference in the row (*p* < 0.05).

**Table 7:** Centrifugation and storage stability of MPP and CCP emulsions prepared by different conditions.

		Emulsion Stability (%)									
		Centrifugation Assay		Storage Assay (W)							
		EA_10_		ES_0_		ES_1_		ES_2_		ES_3_	
		MPP	CCP	MPP	CCP	MPP	CCP	MPP	CCP	MPP	CCP
Pectin concentration	0.5%	46.43	40.71	100.0	81.67	86.67	53.33	83.33	50.00	54.67	48.33
	1.0%	95.00	39.64	100.0	88.33	95.83	53.00	95.00	51.17	94.50	50.67
	1.5%	100	42.14	100.0	100.0	100.0	65.00	100.0	63.00	100.0	60.00
	2.0%	100	41.79	100.0	100.0	100.0	71.67	100.0	66.50	100.0	65.50
pH	3	93.21	41.43	100.0	96.83	100.0	62.67	97.97	59.57	96.58	55.65
	7	44.64	45.00	100.0	70.00	66.80	61.50	57.47	60.23	56.43	58.52
	8	42.14	43.57	100.0	83.33	61.70	69.67	56.83	66.68	54.53	64.38
Oil ratio	10%	93.57	88.33	100.0	100.0	100.0	99.59	100.0	99.49	99.79	99.47
	25%	91.79	51.67	100.0	100.0	98.30	96.90	97.65	71.62	96.65	70.24
	50%	82.86	71.67	100.0	100.0	96.67	98.62	94.52	98.33	92.97	98.24

## References

[1] Ozturk B., McClements D.J. (2016). Progress in natural emulsifiers for utilization in food emulsions. Curr. Opin. Food Sci..

[2] Dickinson E. (2003). Hydrocolloids at interfaces and the influence on the properties of dispersed systems. Food Hydrocoll..

[3] Chivero P., Gohtani S., Yoshii H., Nakamura A. (2014). Physical properties of oil-in-water emulsions as a function of oil and soy soluble polysaccharide types. Food Hydrocoll..

[4] Ma F., Bell A.E., Davis F.J. (2015). Effects of high-hydrostatic pressure and pH treatments on the emulsification properties of gum arabic. Food Chem..

[5] Wang M., Huang B., Fan C., Zhao K., Hu H., Xu X., Pan S., Liu F. (2016). Characterization and functional properties of mango peel pectin extracted by ultrasound assisted citric acid. Int. J. Biol. Macromol..

[6] Chan S.Y., Choo W.S., Young D.J., Loh X.J. (2017). Pectin as a rheology modifier: Origin, structure, commercial production and rheology. Carbohydr. Polym..

[7] Wan L., Chen Q., Huang M., Liu F., Pan S. (2019). Physiochemical, rheological and emulsifying properties of low methoxyl pectin prepared by high hydrostatic pressure-assisted enzymatic, conventional enzymatic, and alkaline de-esterification: A comparison study. Food Hydrocoll..

[8] Yang X., Nisar T., Hou Y., Gou X., Sun L., Guo Y. (2018). Pomegranate peel pectin can be used as an effective emulsifier. Food Hydrocoll..

[9] Schmidt U.S., Schmidt K., Kurz T., Endreß H.U., Schuchmann H.P. (2015). Pectins of different origin and their performance in forming and stabilizing oil-in-water-emulsions. Food Hydrocoll..

[10] Schmidt U.S., Schütz L., Schuchmann H.P. (2017). Interfacial and emulsifying properties of citrus pectin: Interaction of pH, ionic strength and degree of esterification. Food Hydrocoll..

[11] Funami T., Nakauma M., Ishihara S., Tanaka R., Inoue T., Phillips G.O. (2011). Structural modifications of sugar beet pectin and the relationship of structure to functionality. Food Hydrocoll..

[12] Bai L., Huan S., Li Z., McClements D.J. (2017). Comparison of emulsifying properties of food-grade polysaccharides in oil-in-water emulsions: Gum arabic, beet pectin, and corn fiber gum. Food Hydrocoll..

[13] Alba K., Kontogiorgos V. (2017). Pectin at the oil-water interface: Relationship of molecular composition and structure to functionality. Food Hydrocoll..

[14] Liu Z., Pi F., Guo X., Guo X., Yu S. (2019). Characterization of the structural and emulsifying properties of sugar beet pectins obtained by sequential extraction. Food Hydrocoll..

[15] Guo J., Gao Z., Xia J., Ritenour M.A., Li G., Shan Y. (2018). Comparative analysis of chemical composition, antimicrobial and antioxidant activity of citrus essential oils from the main cultivated varieties in China. Food Sci. Technol..

[16] Li Z., Jin R., Yang Z., Wang X., You G., Guo J., Zhang Y., Liu F., Pan S. (2021). Comparative study on physicochemical, nutritional and enzymatic properties of two Satsuma mandarin (*Citrus unshiu* Marc.) varieties from different regions. J. Food Compos. Anal..

[17] Yeoh S., Shi J., Langrish T.A.G. (2008). Comparisons between different techniques for water-based extraction of pectin from orange peels. Desalination.

[18] Singh B., Singh J.P., Kaur A., Singh N. (2020). Phenolic composition, antioxidant potential and health benefits of citrus peel. Food Res. Int..

[19] Kaya M., Sousa A.G., Crépeau M.-J., Sørensen S.O., Ralet M.-C. (2014). Characterization of citrus pectin samples extracted under different conditions: Influence of acid type and pH of extraction. Ann. Bot..

[20] Hu W., Chen S., Wu D., Zhu K., Ye X. (2021). Manosonication assisted extraction and characterization of pectin from different citrus peel wastes. Food Hydrocoll..

[21] Guo X., Zhao W., Liao X., Hu X., Wu J., Wang X. (2017). Extraction of pectin from the peels of pomelo by high-speed shearing homogenization and its characteristics. LWT-Food Sci. Technol..

[22] Blumenkrantz N., Asboe-Hansen G. (1973). New method for quantitative determination of uronic acids. Anal. Biochem..

[23] Peng X.Y., Mu T.-H., Zhang M., Sun H.-N., Chen J.-W., Yu M. (2016). Effects of pH and high hydrostatic pressure on the structural and rheological properties of sugar beet pectin. Food Hydrocoll..

[24] Petkowicz C.L.O., Vriesmann L.C., Williams P.A. (2017). Pectins from food waste: Extraction, characterization and properties of watermelon rind pectin. Food Hydrocoll..

[25] Neckebroeck B., Verkempinck S.H.E., Van Audenhove J., Bernaerts T., de Wilde d’Estmael H., Hendrickx M.E., Van Loey A.M. (2021). Structural and emulsion stabilizing properties of pectin rich extracts obtained from different botanical sources. Food Res. Int..

[26] Xie F., Zhang W., Lan X., Gong S., Wu J., Wang Z. (2018). Effects of high hydrostatic pressure and high pressure homogenization processing on characteristics of potato peel waste pectin. Carbohydr. Polym..

[27] Guo X., Zhao W., Pang X., Liao X., Hu X., Wu J. (2014). Emulsion stabilizing properties of pectins extracted by high hydrostatic pressure, high-speed shearing homogenization and traditional thermal methods: A comparative study. Food Hydrocoll..

[28] Xu D., Zhang J., Cao Y., Wang J., Xiao J. (2016). Influence of microcrystalline cellulose on the microrheological property and freeze-thaw stability of soybean protein hydrolysate stabilized curcumin emulsion. LWT-Food Sci. Technol..

[29] Kurita O., Fujiwara T., Yamazaki E. (2008). Characterization of the pectin extracted from citrus peel in the presence of citric acid. Carbohydr. Polym..

[30] Schmidt U.S., Koch L., Rentschler C., Kurz T., Endreß H.U., Schuchmann H.P. (2015). Effect of Molecular Weight Reduction, Acetylation and Esterification on the Emulsification Properties of Citrus Pectin. Food Biophys..

[31] Lutz R., Aserin A., Wicker L., Garti N. (2009). Structure and physical properties of pectins with block-wise distribution of carboxylic acid groups. Food Hydrocoll..

[32] Asgari K., Labbafi M., Khodaiyan F., Kazemi M., Hosseini S.S. (2020). High-methylated pectin from walnut processing wastes as a potential resource: Ultrasound assisted extraction and physicochemical, structural and functional analysis. Int. J. Biol. Macromol..

[33] Guo X., Han D., Xi H., Rao L., Liao X., Hu X., Wu J. (2012). Extraction of pectin from navel orange peel assisted by ultra-high pressure, microwave or traditional heating: A comparison. Carbohydr. Polym..

[34] Bagherian H., Zokaee Ashtiani F., Fouladitajar A., Mohtashamy M. (2011). Comparisons between conventional, microwave- and ultrasound-assisted methods for extraction of pectin from grapefruit. Chem. Eng. Process. Process. Intensif..

[35] Akhtar M., Dickinson E., Mazoyer J., Langendorff V. (2002). Emulsion stabilizing properties of depolymerized pectin. Food Hydrocoll..

[36] Leroux J., Langendorff V., Schick G., Vaishnav V., Mazoyer J. (2003). Emulsion stabilizing properties of pectin. Food Hydrocoll..

[37] Cui J., Ren W., Zhao C., Gao W., Tian G., Bao Y., Lian Y., Zheng J. (2020). The structure–property relationships of acid- and alkali-extracted grapefruit peel pectins. Carbohydr. Polym..

[38] Zhang L., Ye X., Xue S.J., Zhang X., Liu D., Meng R., Chen S. (2013). Effect of high-intensity ultrasound on the physicochemical properties and nanostructure of citrus pectin. J. Sci. Food Agric..

[39] Ma X., Wang D., Chen W., Ismail B.B., Wang W., Lv R., Ding T., Ye X., Liu D. (2018). Effects of ultrasound pretreatment on the enzymolysis of pectin: Kinetic study, structural characteristics and anti-cancer activity of the hydrolysates. Food Hydrocoll..

[40] Yang J.-S., Mu T.-H., Ma M.-M. (2018). Extraction, structure, and emulsifying properties of pectin from potato pulp. Food Chem..

[41] Nakamura A., Maeda H., Corredig M. (2006). Emulsifying properties of enzyme-digested soybean soluble polysaccharide. Food Hydrocoll..

[42] Ninčević Grassino A., Ostojić J., Miletić V., Djaković S., Bosiljkov T., Zorić Z., Ježek D., Rimac Brnčić S., Brnčić M. (2020). Application of high hydrostatic pressure and ultrasound-assisted extractions as a novel approach for pectin and polyphenols recovery from tomato peel waste. Innov. Food Sci. Emerg. Technol..

[43] Rahmani Z., Khodaiyan F., Kazemi M., Sharifan A. (2020). Optimization of microwave-assisted extraction and structural characterization of pectin from sweet lemon peel. Int. J. Biol. Macromol..

[44] Hu W., Chen S., Wu D., Zhu K., Ye X. (2021). Physicochemical and macromolecule properties of RG-I enriched pectin from citrus wastes by manosonication extraction. Int. J. Biol. Macromol..

[45] Hosseini S., Parastouei K., Khodaiyan F. (2020). Simultaneous extraction optimization and characterization of pectin and phenolics from sour cherry pomace. Int. J. Biol. Macromol..

[46] Rodsamran P., Sothornvit R. (2019). Microwave heating extraction of pectin from lime peel: Characterization and properties compared with the conventional heating method. Food Chem..

[47] Verkempinck S., Kyomugasho C., Salvia-Trujillo L., Denis S., Bourgeois M., Van L., Hendrickx M.E., Grauwet T. (2018). Emulsion stabilizing properties of citrus pectin and its interactions with conventional emulsifiers in oil-in-water emulsions. Food Hydrocoll..

[48] Piriyaprasarth S., Juttulapa M., Sriamornsak P. (2016). Stability of rice bran oil-in-water emulsions stabilized by pectin–zein complexes: Effect of composition and order of mixing. Food Hydrocoll..

[49] Pi F., Liu Z., Guo X., Guo X., Meng H. (2019). Chicory root pulp pectin as an emulsifier as compared to sugar beet pectin. Part 1: Influence of structure, concentration, counterion concentration. Food Hydrocoll..

[50] Jukkola A., Partanen R., Xiang W., Heino A., Rojas O.J. (2019). Food emulsifiers based on milk fat globule membranes and their interactions with calcium and casein phosphoproteins. Food Hydrocoll..

[51] Jamsazzadeh Kermani Z., Shpigelman A., Pham H.T.T., Van Loey A.M., Hendrickx M.E. (2015). Functional properties of citric acid extracted mango peel pectin as related to its chemical structure. Food Hydrocoll..

[52] Jiang Y., Xu Y., Li F., Li D., Huang Q. (2020). Pectin extracted from persimmon peel: A physicochemical characterization and emulsifying properties evaluation. Food Hydrocoll..

[53] Zhao S., Ren W., Gao W., Tian G., Zhao C., Bao Y., Cui J., Lian Y., Zheng J. (2020). Effect of mesoscopic structure of citrus pectin on its emulsifying properties: Compactness is more important than size. J. Colloid Interface Sci..

[54] Koocheki A., Kadkhodaee R., Mortazavi S.A., Shahidi F., Taherian A.R. (2009). Influence of Alyssum homolocarpum seed gum on the stability and flow properties of O/W emulsion prepared by high intensity ultrasound. Food Hydrocoll..

[55] Jafari S.M., Assadpoor E., He Y., Bhandari B. (2008). Re-coalescence of emulsion droplets during high-energy emulsification. Food Hydrocoll..

[56] Alba K., Sagis L.M.C., Kontogiorgos V. (2016). Engineering of acidic O/W emulsions with pectin. Colloids Surf. B Biointerfaces.

[57] Castellani O., Al-Assaf S., Axelos M., Phillips G.O., Anton M. (2010). Hydrocolloids with emulsifying capacity. Part 2—Adsorption properties at the n-hexadecane–Water interface. Food Hydrocoll..

[58] Dickinson E. (2015). Microgels—An alternative colloidal ingredient for stabilization of food emulsions. Trends Food Sci. Technol..

[59] Destribats M., Wolfs M., Pinaud F., Lapeyre V., Sellier E., Schmitt V., Ravaine V. (2013). Pickering Emulsions Stabilized by Soft Microgels: Influence of the Emulsification Process on Particle Interfacial Organization and Emulsion Properties. Langmuir.

[60] Yapo B.M., Robert C., Etienne I., Wathelet B., Paquot M. (2007). Effect of extraction conditions on the yield, purity and surface properties of sugar beet pulp pectin extracts. Food Chem..

[61] Zhang L., Shi Z., Shangguan W., Fang Y., Nishinari K., Phillips G.O., Jiang F. (2015). Emulsification properties of sugar beet pectin after modification with horseradish peroxidase. Food Hydrocoll..

[62] Williams P.A., Sayers C., Viebke C., Senan C., Mazoyer J., Boulenguer P. (2005). Elucidation of the Emulsification Properties of Sugar Beet Pectin. J. Agric. Food. Chem..

[63] Siew C.K., Williams P.A. (2008). Role of Protein and Ferulic Acid in the Emulsification Properties of Sugar Beet Pectin. J. Agric. Food Chem..

